# Systematic Screen for *Drosophila* Transcriptional Regulators Phosphorylated in Response to Insulin/mTOR Pathway

**DOI:** 10.1534/g3.120.401383

**Published:** 2020-06-17

**Authors:** Ying Liu, Jaakko Mattila, Ville Hietakangas

**Affiliations:** * Faculty of Biological and Environmental Sciences, University of Helsinki, Helsinki, 00790, Finland and; ^†^ Institute of Biotechnology, University of Helsinki, Helsinki, 00790, Finland

**Keywords:** phosphorylation, insulin, transcription, growth, mTOR

## Abstract

Insulin/insulin-like growth factor signaling (IIS) is a conserved mechanism to regulate animal physiology in response to nutrition. IIS activity controls gene expression, but only a subset of transcriptional regulators (TRs) targeted by the IIS pathway is currently known. Here we report the results of an unbiased screen for *Drosophila* TRs phosphorylated in an IIS-dependent manner. To conduct the screen, we built a library of 857 V5/Strep-tagged TRs under the control of Copper-inducible metallothionein promoter (pMt). The insulin-induced phosphorylation changes were detected by using Phos-tag SDS-PAGE and Western blotting. Eight proteins were found to display increased phosphorylation after acute insulin treatment. In each case, the insulin-induced phosphorylation was abrogated by mTORC1 inhibitor rapamycin. The hits included two components of the NURF complex (NURF38 and NURF55), bHLHZip transcription factor Max, as well as the *Drosophila* ortholog of human proliferation-associated 2G4 (dPA2G4). Subsequent experiments revealed that the expression of the *dPA2G4* gene was promoted by the mTOR pathway, likely through transcription factor Myc. Furthermore, NURF38 was found to be necessary for growth in larvae, consistent with the role of IIS/mTOR pathway in growth control.

Several aspects of animal physiology, such as tissue growth, metabolism, reproduction, stem cell function and longevity, are regulated in response to nutrients. The insulin/insulin-like growth factor signaling (IIS) pathway is a central regulator of these processes in multicellular animals (Hietakangas and Cohen 2009; Teleman 2009; Partridge *et al.* 2011). Deregulation of IIS is the underlying cause of human diseases, such as diabetes and many types of cancer (Renehan *et al.* 2006; Guo 2014). Due to the conserved nature of IIS, *Drosophila melanogaster* serves as an important genetically tractable model system for the discovery of novel pathway components and targets as well as their physiological roles (Hietakangas and Cohen 2009; Teleman 2009).

IIS regulates cellular functions at different levels, including gene expression. The best-established transcription factor target of *Drosophila* IIS is the Forkhead transcription factor ‘O’ (FoxO), which is phosphorylated by protein kinase AKT, leading to inhibition of FoxO function through cytoplasmic retention (Puig *et al.* 2003; Hietakangas and Cohen 2007). Upon low IIS, activated FoxO promotes the transcription of growth inhibitory genes, such as *4EBP* and *sestrin* (Puig *et al.* 2003; Lee *et al.* 2010). IIS also regulates the phosphorylation of transcriptional cofactors. Phosphorylation of CREB coactivator TORC/CRTC is increased upon insulin treatment through salt-inducible kinase 2 (SIK2), leading to the inhibition of TORC/CRTC activity (Wang *et al.* 2008). This pathway controls starvation resistance and lipid metabolism in adult flies through the central nervous system. A paralog of SIK2, SIK3, phosphorylates histone deacetylase 4 (HDAC4) in response to insulin, thereby inhibiting its deacetylase activity on FoxO (Wang *et al.* 2011). Upon low insulin signaling, HDAC4-mediated deacetylation promotes FoxO activity increasing lipolysis through the Brummer lipase (Wang *et al.* 2011).

The mechanistic target of rapamycin (mTOR) pathway is another important regulator of nutrient-responsive cell physiology. The mTOR pathway integrates several nutrient derived signals, including IIS activity, which promotes mTOR activity (Hietakangas and Cohen 2009). mTOR complex 1 (mTORC1) is an activator of anabolic pathways, such as ribosome biogenesis through all three RNA polymerases (Wullschleger *et al.* 2006). In *Drosophila*, mTORC1 is known to promote the expression of ribosome assembly genes through transcription factor Myc, albeit the molecular mechanism remains poorly understood (Guertin *et al.* 2006; Teleman *et al.* 2008). mTOR also promotes the activities of RNA Pol I and Pol III, which transcribe ribosomal RNAs (rRNAs) and other non-coding RNAs needed for gene expression (Grewal *et al.* 2007; Marshall *et al.* 2012), but the phosphorylation targets of mTOR in this context have remained insufficiently characterized. One such target is chromatin binding protein PWP1, which is phosphorylated in an mTORC1-dependent manner and is necessary for promoting the transcription of rRNAs by RNA Pol I and Pol III (Liu *et al.*, 2018, 2017). TORC1 also phosphorylates transcription factor Reptor, leading to its cytoplasmic retention (Tiebe *et al.* 2015). Upon mTORC1 inhibition by rapamycin, Reptor and its heterodimerization partner Reptor-BP activate gene transcription, controlling the majority of rapamycin-activated genes in *Drosophila* S2 cells.

While several TRs have been identified as phosphorylation targets for IIS/mTOR signaling, comprehensive understanding of the mediators of IIS/mTOR-dependent transcriptional control has not been achieved. Quantitative phosphoproteomics is the state-of-the-art approach for unbiased identification of phosphorylated proteins. Such approach was used to identify 191 proteins, whose phosphorylation changed upon insulin treatment of *Drosophila* S2R+ cells (Vinayagam *et al.* 2016). However, only few transcriptional regulators, such as Jun-related antigen, Modulo and Myb, were among the identified proteins (Vinayagam *et al.* 2016). Therefore, complementary approaches to detect phosphorylation changes in TRs are necessary. Phos-tag SDS-PAGE has emerged as a robust method to separate phosphorylated forms of proteins based on their reduced electrophoretic mobility (Kinoshita *et al.* 2006, 2015). When combined with ectopic expression of proteins of interest, it is possible to overcome the technical limitations posed by low endogenous expression levels. Here we have generated a library of constructs that allow Copper-inducible ectopic expression of 857 Strep/V5-tagged *Drosophila* TRs. Subsequent Phos-tag SDS-PAGE screen was used to identify targets of IIS/mTORC1 signaling.

## Materials AND Methods

### Plasmids, cell culture and transfection

*Drosophila* TRs were cloned into modified pMt-V5-HisA (Invitrogen) vectors by ligase independent cloning (Aslanidis and de Jong 1990). A C-terminal strepIII tag was inserted to the vector backbone, together with a hygromycin resistance gene. The library cDNAs were amplified from the *Drosophila* Genomics Resource Center (DGRC) gold collection library when available. When not in the DGRC collection, TR cDNAs were amplified from 5′RACE (Invitrogen) cDNA library made from adult female flies. Cloning was verified by digestion, the constructs of hits were confirmed by sequencing. *Drosophila* S2 cells were cultured in M3 medium (Sigma) supplemented with 1X Insect Medium Supplement (Sigma), 2% fetal bovine serum (LifeTechnologies) and penicillin/streptomycin (LifeTechnologies). Transfections were performed with Effectene Transfection Reagent kit (Qiagen) according to the manufacturer’s instructions. To analyze phosphorylation, S2 cells were incubated 10 min with 10 μg/ml insulin (Sigma, 16634) alone, or after inhibition for 2 hr with 1 μM Rapamycin (Sigma, 37094) (Liu *et al.* 2017).

### Phos-tag Western blotting

Phos-tag SDS-PAGE and Western blotting were performed according to previous studies (Liu *et al.* 2017). In brief, Phos-tag SDS-PAGE gels were made adding 30 μM Phos-tag reagent according to the manufacturer’s protocol (Wako Chemical). Samples were resolved on SDS-PAGE with Phos-tag and detected via Western blotting mouse anti-V5 (Life Technologies, R960-25) and IRDye 680 goat anti-mouse (LI-COR, 926-32220). Bands were quantified using Image studio lite software (Li-COR). All bands that migrate slower than the most intensive band were considered as putative phosphorylated forms. A small subset of these bands displayed insulin/rapamycin responsiveness.

### Drosophila genetics

Flies were grown at 25°, on medium containing agar 0.6% (w/v), malt 6.5% (w/v), semolina 3.2% (w/v), baker’s yeast 1.8% (w/v), nipagin 2.4%, propionic acid 0.7%. In starvation experiments, larvae were kept on medium contain agar 0.5% (w/v), nipagin 2.5%, and propionic acid 0.7% in PBS, supplemented with or without 5% sucrose (w/v). Fly stocks used in this study were: Tub-Gal4 (Lee *et al.* 1999), Ey-Gal4 (BDSC 5534), *w*^1118^ (BDSC 6326), NURF38 RNAi (BDSC 31341), UAS-Rheb (BDSC, 9688), *mTOR* delP mutant (BDSC 7014), *myc dm*^2^ mutant (Maines *et al.* 2004), UAS-Myc (Johnston *et al.* 1999), UAS-Myc RNAi (VDRC 2947).

### Quantitative RT-PCR

RNA was extracted using a Nucleospin RNA kit (Macherey-Nagel). cDNA was synthesized using SensiFAST cDNA Synthesis kit (Bioline) according to the manufacturer’s protocol. qPCR was performed with a Light cycler 480 Real-Time PCR System (Roche) using SensiFAST SYBR No-ROX qPCR Master Mix (Bioline). The primers used in this research are listed below:

CDK7-F: GGGTCAGTTTGCCACAGTTT

CDK7-R: GATCACCTCCAGATCCGTG

NURF38-F: GTGCCACGTTGGACCAACGCG

NURF38-R: GTAGCCCTTGTGCGGGAAGCAG

NURF55-F: GGACTCTCTTGGAATCCCAACCTC

NURF55-R: GGCCGGTGAAGATGTTCTTGGC

dPA2G4-F: AAGGGCATTGCCTTTCCCAC

dPA2G4-R: GCCTTTAACGTGTAGTCAGCATCG

### Quantification and statistical analysis

Student’s *t*-tests were performed for statistical analyses. n indicates the number of biological replicates and is detailed in the figure legends. Error bars indicate standard deviation.

### Data availability

Plasmids are available upon request. Information of the plasmid library is listed in Supplemental table 1. The authors affirm that all data necessary for confirming the conclusions of the article are present within the article, figures, and tables. Supplemental material available at figshare: https://doi.org/10.25387/g3.12498020.

## Results

### Construction of the epitope tagged library of Drosophila transcriptional regulators

**Figure 1:**
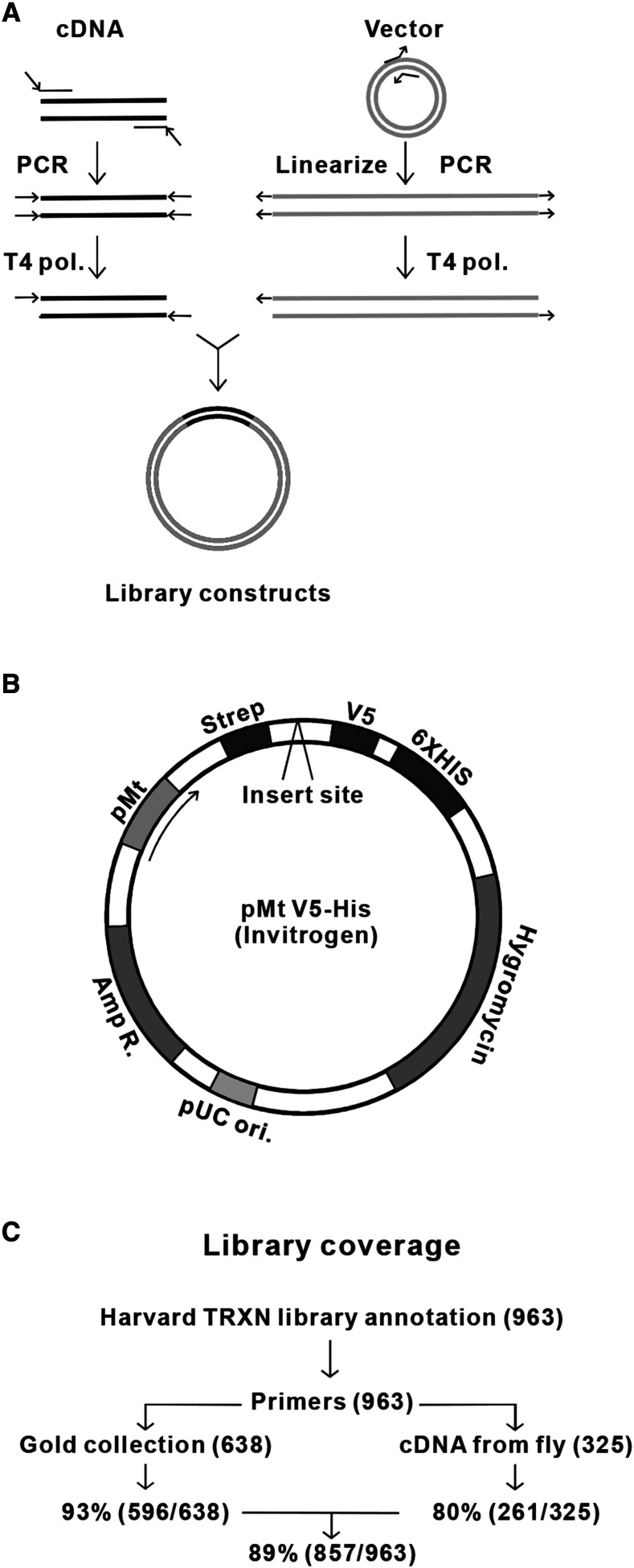
Construction of the library of *Drosophila* transcriptional regulators A. Schematic presentation of ligase-independent cloning of the TRs. B. Schematic map of the vector used. C. Cloning process and coverage of the library.

This study aimed to identify novel transcriptional regulators (TRs) downstream of IIS through a cell-based phosphorylation screen. For this purpose, we constructed an epitope tagged *Drosophila* TR library, based on the annotation by Harvard medical school *Drosophila* RNAi Screening Center (DRSC) ‘Transcription Factors and Related’ RNAi Sub-Library (TRXN) (https://fgr.hms.harvard.edu/drsc-focused-sub-libraries). TRXN includes a set of 963 *Drosophila* genes which are annotated as transcription factor, nuclear protein, and DNA binding protein. The proteins in the library include DNA binding factors, DNA remodeling factors, and histone modifying enzymes along with their associated proteins. Ligase-independent cloning (LIC) was performed to clone these genes into a modified pMt-Strep-V5-HisA expression vector (Figure 1AB). This modified vector was made by inserting a C-terminal Strep-tag and a hygromycin resistance gene to the backbone of the pMt-V5-HisA vector (Figure 1B). The pMt promoter allows Copper-inducible expression of these TRs in cells. Strep-tag is optimal for immobilization and pulldown and V5 is a commonly used epitope tag for antibody detection (*e.g.*, Western blot and immunofluorescence). In total 857 *Drosophila* genes were successfully included in our library, which provides 89% coverage of the TRXN gene set (Figure 1C).

### Screen for TRs downstream of IIS

**Figure 2:**
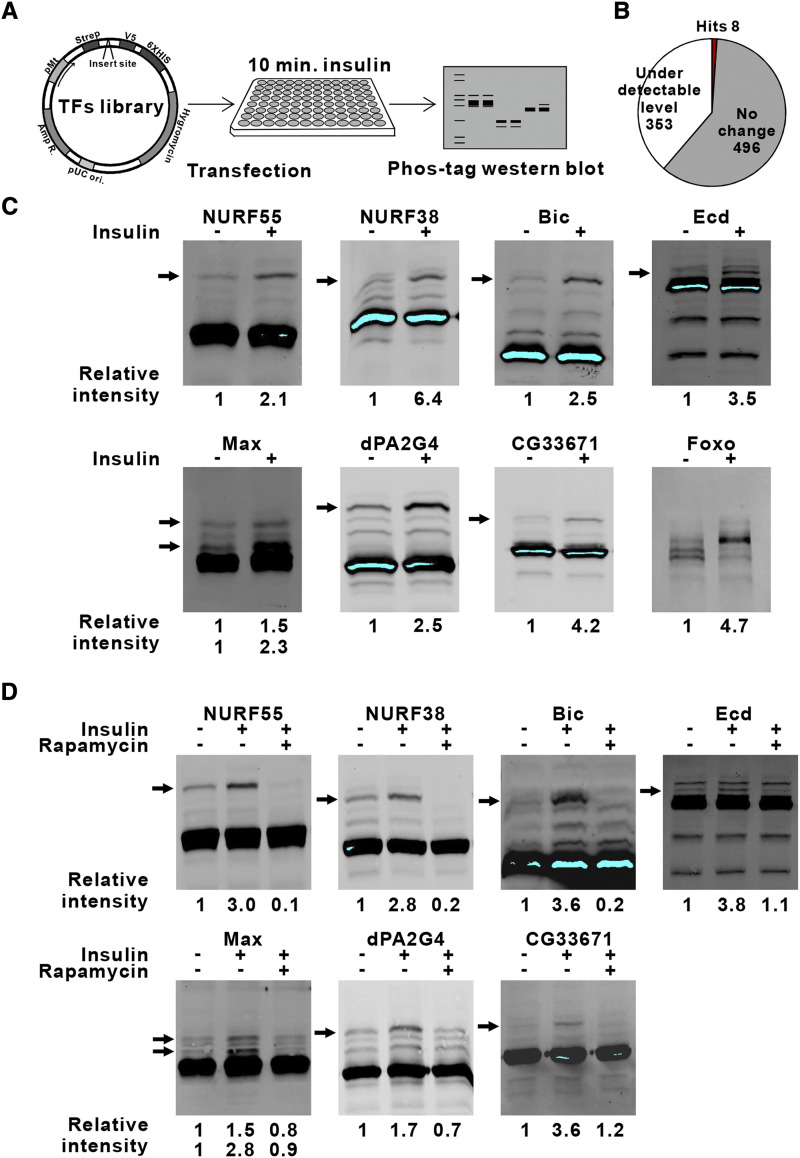
Screen for IIS downstream TRs A. Schematic presentation of the screen via Phos-tag SDS-PAGE and Western blotting. B. Number of detectable TRs (504 out of 857) and number of IIS targeted TRs identified (8 out of 857). C. Immunoblot of S2 cell lysates expressing V5-tagged TRs resolved on Phos-tag SDS-PAGE. Cells were treated 10 min with/without insulin. Insulin responsive bands are indicated by arrows. Relative phosphorylation rates are normalized to unphosphorylated protein species. D. Immunoblot of S2 cell lysates expressing V5-tagged form of TRs resolved on Phos-tag SDS-PAGE. Cells were treated 10 min with insulin alone or in combination with 2 h of rapamycin. Rapamycin responsive bands are indicated by arrows. Relative phosphorylation rates are normalized to unphosphorylated protein species.

Next, we wanted to identify novel TRs regulated by insulin-dependent phosphorylation. To detect changes in phosphorylation status, we utilized the mobility shift of phosphorylated proteins on Phos-tag SDS-PAGE (Kinoshita *et al.* 2006, 2015). Individual TRs were expressed in S2 cells, treated with insulin, or left untreated. Cell lysates resolved on Phos-tag SDS-PAGE were immunoblotted using anti-V5 antibodies (Figure 2A). We were able to detect the expression of 61% of the TRs (504/857) (Figure 2B). In total eight TRs were identified to display increased intensity of a slow migrating band upon insulin treatment, consistent with increased phosphorylation (Figure 2C, Table 1). FoxO was used as a positive control. One of the hits was PWP1, reported earlier (Liu *et al.*, 2017). The Phos-tag SDS-PAGE approach was validated by phosphatase treatment of PWP1, which fully eliminated the insulin-responsive slow migrating bands (Liu *et al.*, 2017). mTOR pathway is a downstream effector of IIS (Hietakangas and Cohen 2009). Hence, we tested whether mTOR pathway is involved in regulating these TRs by using rapamycin, an inhibitor of mTORC1. Indeed, rapamycin treatment fully inhibited the insulin-induced slow migrating band in the case of all TRs (Figure 2D). Interestingly, several of the identified TRs have been reported to control growth in *Drosophila* (Table 1).

**Table 1:** Identified IIS targeted TRs

*Full gene name*	*CG Number*	*Name used in this study*	*mTORC1 dependent*	*Growth regulation*
*Chromatin assembly factor 1*, *p55 subunit*	CG4236	NURF55	Yes	Yes (Wen *et al.* 2012)
*Nucleosome remodeling factor - 38kD*	CG4634	NURF38	Yes	Yes, this study
*bicaudal*	CG3644	Bic	Yes	
*ecdysoneless*	CG5714	Ecd	Yes	Yes (Claudius *et al.* 2014)
*Max*	CG9648	Max	Yes	Yes (Steiger *et al.* 2008)
*CG10576*	CG10576	dPA2G4	Yes	
*Mevalonate kinase*	CG33671	CG33671	Yes	
*no child left behind*	CG6751	dPWP1	Yes	Yes (Liu *et al.* 2017)

### dPA2G4 expression is controlled by mTORC1 and Myc

**Figure 3:**
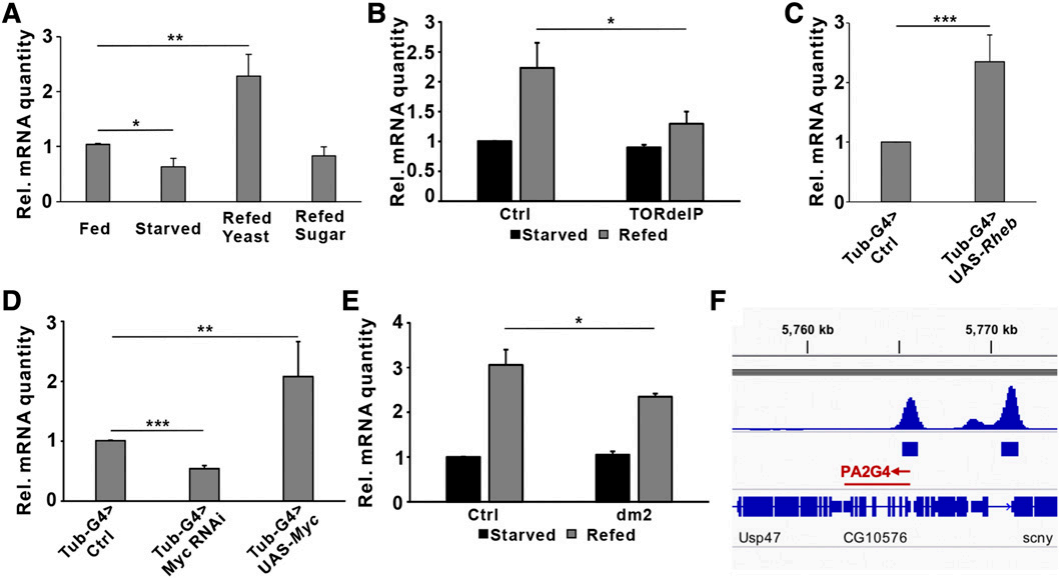
dPA2G4 expression is regulated through IIS/mTORC1 and Myc A. qRT-PCR analysis of *dPA2G4* mRNA expression in third instar larvae upon feeding, or starvation for 24 h (n = 3), or re-feeding with a high-protein diet (20% yeast) for 6 h (n = 3), or a high-sugar diet (20% sucrose) for 6 h (n = 3). *cd**k7* was used as a reference gene. B. qRT-PCR analysis of *dPA2G4* mRNA expression in control (*w*^1118^) (n = 3) and *TOR* mutant (TORdeIP) (n = 3) second instar larvae on 5% sucrose food for 48 h and 20% yeast food re-feeding for 20 h. All samples were normalized to the 5% sugar-starved control larvae. *cd**k7* was used as a reference gene. C. qRT-PCR analysis of dPA2G4 mRNA expression in control (*w*^1118^, tub-Gal4) (n = 3) and Rheb overexpressed (Tub-Gal4) (n = 3) second instar larvae. *cd**k7* was used as a reference gene. D. qRT-PCR analysis of *dPA2G4* mRNA expression in control (*w*^1118^, Tub-Gal4) (n = 3), Myc depletion (Tub-Gal4 > RNAi) (n = 3), and Myc overexpressed (Tub-Gal4 > Myc) (n = 3) second instar larvae. *cd**k7* was used as a reference gene. E. qRT-PCR analysis of *dPA2G4* mRNA expression in control (*w*^1118^) (n = 3) and *myc* mutant (*dm*^2^) (n = 3) second instar larvae on 5% sucrose food for 48 h and 20% yeast food re-feeding for 20 h. All samples were normalized to the 5% sugar-starved control larvae. *cd**k7* was used as a reference gene. F. modERN ChIP-seq data (ENCSR191VCQ; modencode:5008; ChIP-seq against Myc in *D. melanogaster* wandering third instar larvae) displayed a significant enrichment of Myc chromatin binding close to *dPA2G4* transcriptional start site. ∗*P* < 0.05, ∗∗*P* < 0.01, ∗∗∗*P* < 0.001 by Student’s *t*-test. Error bars indicate SDs.

One of the hits was CG10576, which encodes the *Drosophila* ortholog of human proliferation-associated 2G4 (PA2G4) and will be referred to as *Drosophila* proliferation-associated 2G4 (dPA2G4). Downstream targets of nutrient sensing pathways are often regulated transcriptionally in parallel to posttranslational regulation. For example, our previous data showed that PWP1 is regulated by nutrients and IIS/mTOR pathway through both phosphorylation and gene expression (Liu *et al.*, 2017). Thus, we wanted to test, whether *dPA2G4* gene expression is also nutrient regulated *in vivo*. Indeed, *dPA2G4* mRNA levels were downregulated in fasting larvae, while being prominently elevated upon re-feeding with protein-rich yeast diet, but not similarly induced by sugar-only diet (Figure 3A). Next, we asked whether this expression regulation is dependent on the mTOR pathway. Indeed, the protein-induced expression of *dPA2G4* was blunted in *mTOR* mutant larvae (Figure 3B). Moreover, activation of mTORC1 pathway by overexpressing *Ras homolog enriched in brain* (*Rheb*) promoted *dPA2G4* expression (Figure 3C). These data suggest *dPA2G4* expression is controlled through the mTORC1 pathway.

The transcription factor Myc is an established downstream effector of the IIS/mTORC1 pathway (Teleman *et al.* 2008). To address whether Myc is responsible for the transcriptional regulation of *dPA2G4*, we analyzed *dPA2G4* expression levels in larvae following *myc* loss- and gain-of-function. *dPA2G4* expression was significantly upregulated upon Myc overexpression and reduced upon depletion of Myc (Figure 3D). In addition, induction of *dPA2G4* mRNA expression by protein-rich diet was partially prevented in *myc* mutant larvae (Figure 3E), implying that Myc contributes to nutrient-responsive expression of *dPA2G4*. Moreover, *in si**lic**o* analysis of modENCODE ChIP-seq data (ENCSR191VCQ) in wandering 3^rd^ instar larvae revealed a significant enrichment of Myc chromatin binding close to the transcriptional start site of *dPA2G4* (Figure 3F). In conclusion, our data indicates that transcription of *dPA2G4* is promoted by mTORC1 pathway through transcription factor Myc in response to protein-rich diet.

### NURF38 is necessary for animal growth

**Figure 4:**
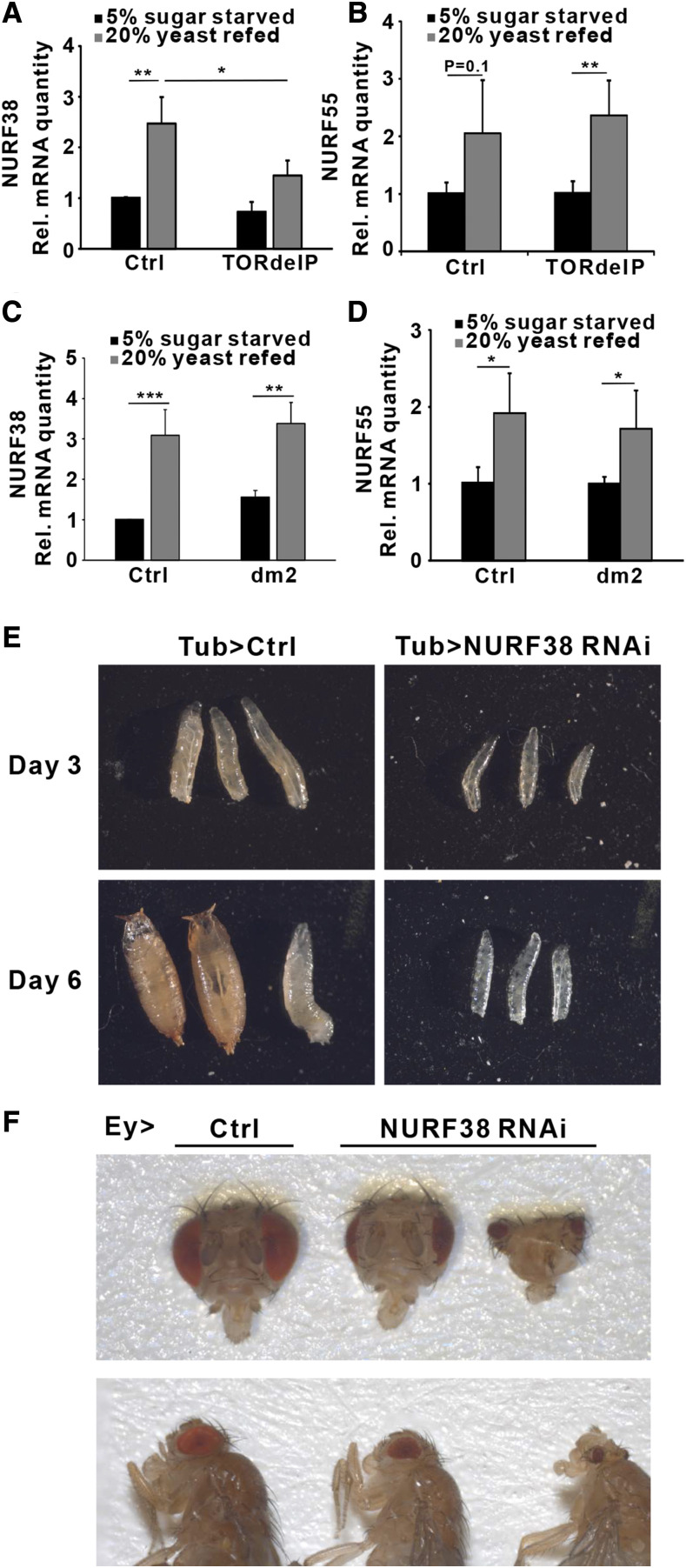
NURF complex is a nutrient-dependent growth regulator AB. qRT-PCR analysis of *NURF38* and *NURF55* mRNA expression in control (*w*^1118^) (n = 3) and *TOR* mutant (TORdeIP) (n = 3) second instar larvae on 5% sucrose food for 48 h and 20% yeast food re-feeding for 20 h. All samples were normalized to the 5% sugar-starved control larvae. *cd**k7* was used as a reference gene. CD. qRT-PCR analysis of *NURF38* and *NURF55* mRNA expression in control (*w*^1118^) (n = 3) and *myc* mutant (*dm*^2^) (n = 3) second instar larvae on 5% sucrose food for 48 h and 20% yeast food re-feeding for 20 h. All samples were normalized to the 5% sugar-starved control larvae. *cd**k7* was used as a reference gene. E. Representative images of control and NURF38 depleted (Tub-Gal4) larvae and pupae. F. Representative images of control (*w*^1118^) and NURF38 eye (Ey-Gal4)-depleted adult eye. ∗*P* < 0.05, ∗∗*P* < 0.01, ∗∗∗*P* < 0.001 by Student’s *t*-test. Error bars indicate SDs.

Our Phos-tag data revealed that two subunits of the ATP-dependent chromatin remodeling complex NURF, NURF38 and NURF55 (Caf1-55), are phosphorylated upon insulin treatment, in mTORC1-dependent manner (Figure 2CD). Similar to *dPA2G4*, mRNA levels of *NURF38* were significantly increased in response to re-feeding with a protein rich diet, and this elevated expression was partially blunted in *mTOR* mutant larvae (Figure 4A). The expression of *NURF55* was also elevated upon yeast re-feeding, but this induction was insensitive to loss of mTOR (Figure 4B). In contrast to *dPA2G4*, *NURF38* and *NURF55* expression levels were not affected in *myc* mutants (Figure 4 CD). Thus, the NURF38 expression is regulated in response to nutrients through mTOR pathway, but is independent of Myc.

A previous study has shown that *Drosophila* mutants of *NURF55* display a strong undergrowth phenotype (Wen *et al.* 2012). To explore the possible role of NURF38 in growth regulation, we depleted NURF38 ubiquitously in *Drosophila* larvae by Tub-GAL4 > NURF38 RNAi. The NURF38 depleted larvae remained viable for several days, but failed to increase in size, consistent with impaired tissue growth (Figure 4E). Moreover, eye-specific depletion of NURF38 using Ey-GAL4 led to strongly reduced eye size, suggesting a tissue autonomous role for NURF38-dependent growth regulation (Figure 4F).

## Discussion

Animals coordinate their growth rate and metabolism in a changing nutrient landscape, which requires dynamic regulation of gene expression. Therefore, it is important to understand the identity and roles of the transcriptional regulators (TRs) involved. Here, we identify several TRs with a putative role in nutrient-dependent gene regulation. Specifically, we (1) identify two components of the NURF complex, Max and dPA2G4 as phosphorylation targets of IIS/mTORC1 signaling in *Drosophila* S2 cells; (2) provide evidence that expression of *dPA2G4* is regulated by IIS/mTORC1 signaling, likely through Myc; (3) identify *NURF38* as a necessary gene for tissue growth in larvae; (4) construct a library of 857 V5/Strep-tagged TRs compatible for Copper-induced expression in *Drosophila* cells. Collectively, this study demonstrates the potential of Phos-tag SDS-PAGE for unbiased identification of new phosphorylation targets of signaling pathways. Thus, it can be used to complement other approaches, such as phosphoproteomics. Some technical limitations were also observed. >350 proteins remained undetected, including Reptor (a known target of mTORC1). This might be due to high protein turnover or cell lethality or caused by technical reasons, such as insufficient transfer of large proteins or poor transfection efficiency. It should be also noted that some of the genes included in the library might be misannotated as TRs, including some of the hits (*e.g.*, CG33671/*Mevalonate kinase*).

The *Drosophila* nucleosome remodeling factor (NURF) complex is a conserved ATP-dependent chromatin remodeling complex with four subunits: NURF38, NURF55, NURF301 and ISWI (Becker and Hörz 2002; Corona and Tamkun 2004). The NURF complex was initially discovered as a regulator of heat shock gene expression, a key mechanism to maintain cellular proteostasis (Tsukiyama and Wu 1995). Moreover, NURF55, one of the discovered IIS/mTOR phosphorylation targets, has been shown to be necessary for larval growth (Wen *et al.* 2012) as well as for regulation of lipid droplet size in the larval fat body (Yao *et al.* 2018). We found that another component of the NURF complex, NURF38, is necessary for larval growth along with its IIS/mTOR-dependent phosphorylation. Notably, however, mutants of *NURF301* do not display a strong undergrowth phenotype, but an ecdysone-dependent pupariation phenotype (Badenhorst *et al.* 2005). Thus, it remains to be determined whether growth regulation by NURF38 and NURF55 is mediated through their function in the NURF complex and how they contribute to the gene regulatory effects of IIS/mTOR signaling.

Ribosome biogenesis is a key process in regulating cellular growth capacity and it is tightly coordinated by the IIS/mTOR pathway. Ribosome biogenesis involves all three RNA polymerases and is likely regulated through multiple effectors. Myc is known to control ribosome biogenesis downstream of mTOR signaling, as inhibition of mTORC1 leads to downregulation of Myc protein levels (Teleman *et al.* 2008). However, the mechanistic details of how mTORC1 signaling regulates Myc have remained unresolved. Interestingly, our screen identified the Myc heterodimerization partner Max as a phosphorylation target by mTORC1. Earlier studies in mammals have shown that Max is a phosphoprotein, constitutively phosphorylated by CKII on N-terminal serines S2 and S11 (Berberich and Cole 1992; Koskinen *et al.* 1994). Future studies will be needed to address whether Max phosphorylation contributes to the mTORC1-dependent regulation of Myc-Max target genes in *Drosophila*. Another downstream target of mTORC1 signaling is PWP1, which is phosphorylated in an mTORC1-dependent manner and necessary for RNA Pol I and III-dependent expression of ribosomal RNA (Liu *et al.* 2017, 2018). In addition to phosphorylation, *PWP1* gene expression is promoted by IIS/mTOR signaling through Myc transcription factor (Liu *et al.* 2017). Interestingly, we demonstrated here that dPA2G4 is under similar dual regulation by phosphorylation and gene expression. In mammalian cells, PA2G4 has been found to localize to the nucleolus, bind to Pol I, and promote rRNA expression (Karlsson *et al.* 2016; Nguyen *et al.* 2016). It can be therefore hypothesized that PA2G4 is a candidate to cooperate with PWP1 in regulating nutrient-responsive ribosome biogenesis *in vivo*.
